# Specificity of psychopathology across levels of severity: a transdiagnostic network analysis

**DOI:** 10.1038/s41598-019-54801-y

**Published:** 2019-12-04

**Authors:** Robin N. Groen, Marieke Wichers, Johanna T. W. Wigman, Catharina A. Hartman

**Affiliations:** University of Groningen, University Medical Center Groningen, Interdisciplinary Center Psychopathology and Emotion regulation, Groningen, The Netherlands

**Keywords:** Epidemiology, Human behaviour, Comorbidities

## Abstract

A prominent hypothesis within the field of psychiatry is that the manifestation of psychopathology changes from non-specific to specific as illness severity increases. Using a transdiagnostic network approach, we investigated this hypothesis in four independent groups with increasing psychopathology severity. We investigated whether symptom domains became more interrelated and formed more clusters as illness severity increased, using empirical tests for two network characteristics: global network strength and modularity-based community detection. Four severity groups, ranging from subthreshold psychopathology to having received a diagnosis and treatment, were derived with a standardized diagnostic interview conducted at age 18.5 (n = 1933; TRAILS cohort). Symptom domains were assessed using the Adult Self Report (ASR). Pairwise comparisons of the symptom networks across groups showed no difference in global network strength between severity groups. Similar number and type of communities detected in the four groups exceeded the more minor differences across groups. Common clusters consisted of domains associated with attention deficit hyperactivity disorder (ADHD) and combined depression and anxiety domains. Based on the strength of symptom domain associations and symptom clustering using a network approach, we found no support for the hypothesis that the manifestation of psychopathology along the severity continuum changes from non-specific to specific.

## Introduction

Onset of mental ill health is often a long and gradual process^1^. Longitudinal findings indicate that symptoms below the clinical range frequently precede later clinical psychopathology^2^, suggesting that there might not be a clear-cut threshold between the absence and presence of a disorder. It is clear that *quantitative* differences in the manifestation of psychopathology exist for individuals below the clinical range as compared to those experiencing full-blown clinical disorders. However, questions remain about the *type* of symptom manifestation at different levels of illness severity. In particular, it is unclear whether the structure in which symptoms relate to one another is altered as illness severity increases.

A plausible hypothesis is that symptom expression becomes increasingly specific along the unfolding course of illness severity^3^. In the so-called clinical staging literature, early stage psychopathology is frequently described as non-specific, mixed symptoms that lack sufficient severity or clarity to justify a diagnosis^4–6^. In this view, early symptoms are pluripotent, meaning that initial diffuse symptom presentation may develop into various illness trajectories leading up to specific syndrome clusters^3^. This idea aligns with the hypothesis of increasing differentiation from a broader psychopathology dimension to more distinct presentations of psychopathology over the course of development from childhood into adolescence into adulthood^7–9^. Findings were not definite; Sterba and colleagues found that psychopathology consistently manifested as distinct dimensions over development^9^, while other designs suggested that both increased specificity and increased differentiation of psychopathology may take place^7,8^. Note however that these studies did not address the process of increasing illness severity as outlined in the clinical staging model. Following this analogy - stages reflecting points on the psychopathology continuum^4^ – it is possible to examine whether the manifestation of psychopathology changes along the continuum by comparing individuals situated at different illness severity levels.

To investigate and compare symptom interrelations at different stages of illness severity, the optimal study design and method of analysis addresses the following three issues. First, due to the hypothesized pluripotent and non-specific nature of early symptomatology, we need to take a transdiagnostic approach: including a broad range of symptoms spanning multiple psychopathological syndrome domains. Second, an implication of a broad symptom range and the possibility that psychopathology manifests as non-specific when illness severity is low is that symptoms may be interrelated in many possible combinations. An analytic approach that addresses this is the network approach, which is not aimed at data-reduction through positing a smaller number of latent variables as in more traditional methods like factor analysis, but brings all possible relations at the symptom level itself to the foreground^10^. A network approach allows for investigation of symptom clustering through the identification of so-called communities, i.e., subgroups of symptoms that are more interrelated with each other than with the rest of symptom domains in the network). The presence of specific communities would be consistent with the hypothesized, more articulate, manifestation of psychopathology when illness-severity is high. Third, our research question ‘to examine alterations in interrelations between symptoms along the continuum of illness severity’ is complicated by the fact that individuals lower on this continuum have lower symptom severity as compared to individuals higher on the continuum. Differences in symptom severity alter the connection strengths between nodes in the network because they influence the symptom variance^11^, and therefore the covariance among symptoms, which is the unit of analysis in networks. Whether symptom interrelations change with increasing severity can only be studied without the possible confound of this ‘severity aspect’. Therefore, we need to eliminate the differences in symptom variance to obtain a structure of interrelations that is not merely due to symptom severity. Comparable variances at different levels of illness severity can be achieved through use of different severity thresholds that determine whether a symptom domain is endorsed or not.

## Aims of the Study

In the present study, we used data from the Tracking Adolescents Individuals Lives Survey (TRAILS) study to investigate whether psychopathology manifest as more specific with increasing illness severity. We evaluated specificity by modeling conditionally independent associations between symptoms using network analysis. When symptoms are interrelated in equal measure, which is implied by non-specificity, there is only shared variance among them and the network reveals that symptoms are *not* uniquely associated to one another. In contrast, when there is specificity, symptoms are uniquely associated to one another, which becomes visible in denser networks. To assess whether more specific interrelations emerge with increasing illness severity, we compared four frequently observed severity groups (subthreshold, past clinical symptoms, current clinical symptoms, and current clinical symptoms combined with referral to outpatient clinics), which functioned as proxies for different severity stages that are central in the staging model and reflect points on the psychopathology continuum. Using empirical tests for two network characteristics: global network strength and community detection, we investigated symptom interrelations in two steps. First, we estimated global network strength, which summarizes the amount of unique explained variance (i.e. on top of the shared variance), and thus provides a global estimate of symptom relations, which we hypothesize will become greater with increased illness severity. In a second step, to obtain a more detailed picture of the structure of the network, we used modularity-based community detection to compare the extent and type of symptoms clustering together. We hypothesize that more communities will be detected and that these resemble specific psychopathology dimensions with increasing illness severity.

## Method

### Participants

Data came from the fourth wave (T4) of the Tracking Adolescents Individual Lives Survey (TRAILS) study. TRAILS is an ongoing, prospective cohort study with bi- or triennial assessments investigating the causes and consequences of mental ill-health from pre-adolescence into adulthood in a population based cohort (TRAILS PC) and a clinically referred cohort (TRAILS CC).

Data was pooled from both cohorts, to ensure a wide range of problem severity (i.e., both subclinical and clinical levels of psychopathology). A combined 2303 participants (1881 in TRAILS PC and 422 in TRAILS CC) completed T4. The Dutch Central Committee on Research Involving Human Subjects (CCMO) approved TRAILS, and all participants provided informed consent. Participants received a gift certificate (10 euro) for participation. Descriptions of survey design and data collection procedures are available in detail elsewhere^12^, and summarized in the Supplementary Materials.

### Design

At T4, participants completed a wide variety of measures (complete overview reported elsewhere^12^) including the Adults Self Report^13^ and were invited for a diagnostic interview; the Composite International Diagnostic Interview^14^. All respondents (N = 1933) who completed the CIDI were selected for the current study. Participants were allocated to severity subgroups based on their CIDI diagnostic status (see below for more details). Network analysis was used to map symptom interrelations for each severity group separately. In these networks, nodes consisted of symptom domain-specific aggregates of ASR items (see section “symptom domains” below for more details).

### Diagnostic assessment

#### CIDI

Trained lay interviewers conducted the computer-assisted CIDI to obtain lifetime and 12-month (12mo), DSM-IV-based^15^ diagnoses. The CIDI starts with a screening questionnaire. In case of a positive screen, the interviewer administered the corresponding disorder-specific section to assess which DSM-IV criteria were met, what the timing of the disorder was and whether the individual had received treatment by a health care professional for their symptoms. Included diagnoses were mood disorders, anxiety disorders, behavioral disorders, substance use disorders and pathological gambling. A detailed list of diagnoses can be found in the Supplementary Materials.

#### Severity groups

For the purpose of this study, four severity groups were formed using the information obtained during the diagnostic assessment (CIDI). The least severe group (1) called ‘CIDI subthreshold’, contained participants from TRAILS PC who experienced subthreshold psychopathology (see *subthreshold disorders* below for more details on this category). Participants from TRAILS PC who met criteria for a CIDI lifetime diagnosis, but did not have a diagnosis during the past year (CIDI 12-month diagnosis) were considered to have slightly more severe current (i.e., six-month period that ASR assesses) psychopathology than the subthreshold group. These participants formed group (2) ‘CIDI lifetime only’. Participants from TRAILS PC who met criteria for CIDI 12-month diagnosis, but who indicated not to have received treatment, formed group (3) ‘CIDI 12-month diagnosis no-treatment’. Participants from TRAILS PC with a CIDI 12-month diagnosis who received treatment (i.e., responded affirmative to CIDI treatment question) and participants from TRAILS CC with a CIDI 12-month diagnosis together formed group (4) ‘CIDI 12-month diagnosis with treatment’. This group was considered to experience the most severe current (past six months) psychopathology, based on the frequently reported positive associations between the probability of service use and symptom severity^16,17^. To ascertain that these groups indeed represented increasing levels of illness severity, we compared them on the Adult Self Report total score.

#### Subthreshold disorders

For all full-syndrome disorders assessed with CIDI, we also assessed subthreshold status. Except for minor depression and recurrent brief depression, no official subthreshold definitions exist. Following the proposed rule by Roberts and colleagues^18^, we defined subthreshold disorder as follows. If a minimum number of symptoms was required to meet a DSM-IV criterion, half the symptoms were needed for the subthreshold condition. Similarly, if a minimum length of duration was specified half the duration was needed for the subthreshold condition. For disorders with both a minimal duration and minimum number of symptoms criterion, either at least half the duration and full symptom number or full duration and at least half the symptoms was required. For disorders without criteria specifying a number of symptoms, more than half of the criteria needed to be fulfilled, of which criteria A was mandatory, as this is the defining criterion for all disorders studied here. In Supplementary Table S1, disorders are listed with their definitions, as well as their use in previous work.

### Symptom domains

#### ASR

We assessed symptomatology (during the past six months) with the ASR. With 123 items, the ASR covers a broad range of emotional and behavioral problems that participants rate with ‘0 = not true’, ‘1 = somewhat or sometimes true’, or ‘2 = very or often true’. We aggregated multiple ASR items (see Supplementary Table S2 for the individual items) to derive symptom domains as described in DSM categories. We included the following domains: core depressive symptoms, negative cognitions about oneself, worrying, fearfulness, fatigue, substance use, medically unexplained pain, emotion dysregulation, attention problems, hyperactivity, and impulsiveness.

### Severity threshold

We aimed to compare the structure of symptom interrelations between groups differing in illness severity, without confounding by these differences in severity. Differences in variance are associated with severity differences (such as floor effects in healthy groups and ceiling effects in severe groups) directly influence network connections^11^. With a different severity threshold (see Supplementary Table S2 for details) for each group indicating whether an individual did (=1) or did not (=0) experience the symptom domain, we enforced symptom domains to have comparable prevalences in each group. The highest thresholds were applied to group 4, representing individuals with the most severe psychopathology, followed by decreasing thresholds in groups 3, 2, and 1, respectively.

## Statistical Analysis

### Network estimation

We estimated symptom domain networks, using the Ising model^19^, a pairwise Markov Random Field model appropriate for binary data^20^. This model was estimated using the R-package *IsingFit*^20^, in which a combination of L1-regularized logistic regressions and model selection based on the Extended Bayesian Information Criterion (EBIC) is implemented to derive the conditionally dependent pairwise associations (edges) between symptom domains. Edges that after regularization are not set to zero are considered sufficiently strong to be included in the model. Further details on this model and model estimation are provided in the Supplementary Materials. Additionally, we performed network stability analyses using the R-package *bootnet*^21^ to examine the accuracy of the edge weights through bootstrapped (2500 iterations) 95% confidence intervals (CIs) and appended the results as Supplementary Material of this article. Because we observed wide edge CIs, we repeated all our analyses using unweighted networks (edges absent ‘0’ or present ‘1’) as a robustness check. These results can be found in the Supplementary Materials. Networks were visualized using the R-package *qgraph*^22^. We used Spearman correlations to estimate the structural similarity between each pair of networks^23^. For the unweighted networks, we computed the Jaccard Index^24^ for each network comparison using the R-package *BiRewire*^25^.

### Network comparison

#### Global strength

To examine whether subthreshold psychopathology manifests as non-specific, meaning that symptoms interrelate in equal measure and do not uniquely associate with one another, we compared the groups in terms of global strength. Global strength is operationalized as the absolute sum of all edges in the network, and thus reflects the sum of unique variance in the network. When symptom interrelations are more specific, as we hypothesized in case of higher illness severity, more unique variance is present, resulting in higher global strength. We used the permutation testing procedure implemented in the R-package *NetworkComparisonTest*^26^ to statistically assess the difference in global network strength, between groups. In this procedure, observed differences between groups are compared to a permutation distribution that is obtained by randomly regrouping individuals 10 000 times and calculating the difference score between groups each time. Currently it is not possible to compare multiple groups using the NCT. Hence, we performed six pairwise comparisons for global network strength.

#### Community detection

To evaluate differences in symptom domain clustering at the different severity levels, we used modularity-based community detection algorithms. A community structure is present when there are at least two clusters identified that feature symptom-domains with strong within-cluster interrelations, and sparse associations with other symptom domains in the network. Observing no community structure indicates that there are no preferential interrelations between symptom domains, and is thus a sign that manifestation of psychopathology is non-specific. We expected to observe no community structure in the subthreshold group. With increasing illness severity, we expected more communities to arise. We used two algorithms^27,28^ to ensure stability of detecting the community structure across methods. Both are implemented in the R package *igraph* package^29^, and have previously been used to identify community structures in psychopathological data^30,31^. Both algorithms (albeit using different methods, see supplement for more details) partition the network in subnetworks (communities) until the so-called modularity index Q is optimized. Q is calculated as the difference between the edge strengths within the assigned communities of the original network, and the expected edge strengths within the same communities when the network would be random. If a value of *Q* = 0 is returned this indicates that no division in subnetworks exist and no community structure is obtained. Generally modularity values greater than 0.30 are considered a clear indication of communities^32^. We expected increasing values of Q with increasing psychopathology severity. We compared groups in which community structures were present on the number of communities and the type of symptom domains clustering.

### Ethical standards

The authors assert that all procedures contributing to this work comply with the ethical standards of the relevant national and institutional committees on human experimentation and with the Helsinki Declaration of 1975, as revised in 2008.

## Results

### Sample

Wave 4 was completed by *N = *2303 individuals (TRAILS PC and CC pooled), of which *N = *1863 completed both the ASR and the CIDI. Individuals without subthreshold, lifetime or past year psychopathology (N = 561), and with partially missing data on any of the 11 symptom domains (N = 11) were excluded from the current study. Characteristics of the individuals in the remaining four groups are shown for each group separately in Table 1.

In terms of group composition based on the CIDI, most noticeable differences were the following: percentages of disruptive disorders were twice as high in group 1 (subthreshold) and group 2 as compared to group 3 (past year no treatment) and group 4 (past year received treatment). Percentages of mood disorders and ADHD were highest in the group 4.

Although our distinction in four groups based on the CIDI was on global severity criteria, mean differences between groups on the ASR total score (note: before severity thresholds to ASR items were applied) (see Table 1) confirmed that groups differed in psychopathology severity. Likewise, the mean scores on each symptom domain (listed in Table 1) also indicated severity differences between groups, with low symptom domain scores for the least severe group and the highest scores in the most severe group. After applying the severity correction, prevalences (listed in Supplementary Table S3) were comparable across groups. Tests of equality of variance indicated that groups did not significantly differ in symptom domain variances (results can be found as Supplementary Table S4).

**Table 1:** Demographic and clinical characteristics of the four severity groups based on CIDI.

	CIDI subthreshold (n = 492)	CIDI lifetime only (n = 205)	CIDI 12mo-no- treatment (n = 291)	CIDI 12mo-yes- treatment (n = 303)	df	Test statistics	p-value
Female (%)	47.9_a_	53.9_a,b_	60.1_b_	60.2_b_	3	χ = 14.7	0.002
Age (M ± SD)	18.52_a_ (0.61)	18.51_a_ (0.62)	18.57_a_ (0.60)	18.58_a_ (0.65)	3	F = 1.0	0.384
**Social Economic Status (SES) (%)**							
*Lowest 25% SES*	16.5_a_	24.9_a,b_	28.4_b_	29.1_b_	6	χ = 23.3	P < 0.001
*Middle 50% SES*	53.0_a_	49.2_a_	49.8_a_	51.9_a_			
*Highest 25% SES*	30.5_a_	25.9_a,b_	21.8_a,b_	19.0_b_			
**Diagnostic categories (%** ^1^ **)**							
*Anxiety disorders*	68.5^#^	53.7	57.0	58.4			
*Attention Deficit Hyperactivity Disorder*	0.8^#^	4.4	6.9	15.8			
*Disruptive disorders*	28.5^#^	28.8	15.8	13.5			
*Mood disorders*	25.4^#^	31.2	22.3	40.9			
*Substance use disorders*	18.3^#^	4.9	16.2	15.2			
Comorbidity > 1 subthreshold or threshold diagnosis (%)	44.1	27.8	35.0	46.5			
Adult Self Report total score (M ± SD)	25.02 (16.97)	34.90 (22.13)	42.6 (24.60)	51.9 (27.33)	3	F = 99.2	P < 0.001
*Clinical range (%)*	4.5_a_	13.2_b_	20.3_b_	30.0_c_	6	χ = 155.6	P < 0.001
*Borderline range (%)*	6.5_a_	12.7_b_	12.4_b_	20.1_b_			
*Healthy range (%)*	89.0_a_	74.1_b_	67.4_b_	49.8_c_			
**Symptom domains (M ± SD):**							
*Core depression*	0.59_a_ (1.02)	0.89 _b_ (1.13)	1.16_b_ (1.34)	1.61_c_ (1.53)	3	F = 44.0	P < 0.001
*Negative cognitions about oneself*	0.87_a_ (1.22)	1.44_b_ (1.57)	1.71_b_ (1.78)	2.34_c_ (2.05)	3	F = 53.1	P < 0.001
*Fatigue*	0.74_a_ (1.04)	1.00_a_ (1.07)	1.32_b_ (1.23)	1.68_c_ (1.32)	3	F = 44.0	P < 0.001
*Fearfulness*	0.54_a_ (0.77)	0.78_b_ (0.94)	0.92_b_ (0.94)	1.22_c_ (1.16)	3	F = 34.4	P < 0.001
*Medically unexplained pain*	0.27_a_ (0.79)	0.39_a_ (1.07)	0.74_b_ (1.43)	1.10_c_ (1.47)	3	F = 34.5	P < 0.001
*Worrying*	2.04_a_ (1.70)	2.69_b_ (1.84)	3.00_b_ (1.91)	3.73_c_ (2.01)	3	F = 54.4	P < 0.001
*Impulsivity*	1.07_a_(1.19)	1.49_b_(1.35)	1.69_b_,_c_(1.48)	1.96_c_(1.51)	3	F = 30.1	P < 0.001
*Attention problems*	3.40_a_ (2.18)	3.88 _a_ (2.21)	4.77 _b_(2.54)	5.40 _c_(2.59)	3	F = 50.7	P < 0.001
*Hyperactivity/restlessness*	1.15_a_ (1.12)	1.60_b_ (1.40)	1.90_b,c_ (1.39)	2.17_c_ (1.55)	3	F = 41.8	P < 0.001
*Emotion dysregulation*	0.83_a_ (1.06)	1.19_b_ (1.29)	1.39_b_ (1.34)	1.67_c_ (1.37)	3	F = 31.2	P < 0.001
*Substance use*	0.55_a_ (0.82)	0.65_a,b_ (0.88)	0.83_b_ (1.03)	0.76_b_ (1.0)	3	F = 6.5	P = 0.001
**Number of symptom domains endorsed (%)**							
0	41.7_a_	50.6_a_	45.0_a_	46.4_a_	6	χ = 13.3	P = 0.04
1	26.6_a_	17.1_b_	24.4_a,b_	18.2_b_			
*2 or more*	31.7_a_	32.2_a_	30.6_a_	35.4_a_			

Each subscript letter denotes a subset of the severity groups whose column proportions do not differ significantly from each other at the *p* = 0.05 level.

1. Note: percentages reflect the proportion of individuals meeting criteria for at least one disorder from this category.

^#^Note: this reflects the % of individuals meeting subthreshold status for this diagnostic category.

### Estimated group networks

**Figure 1:**
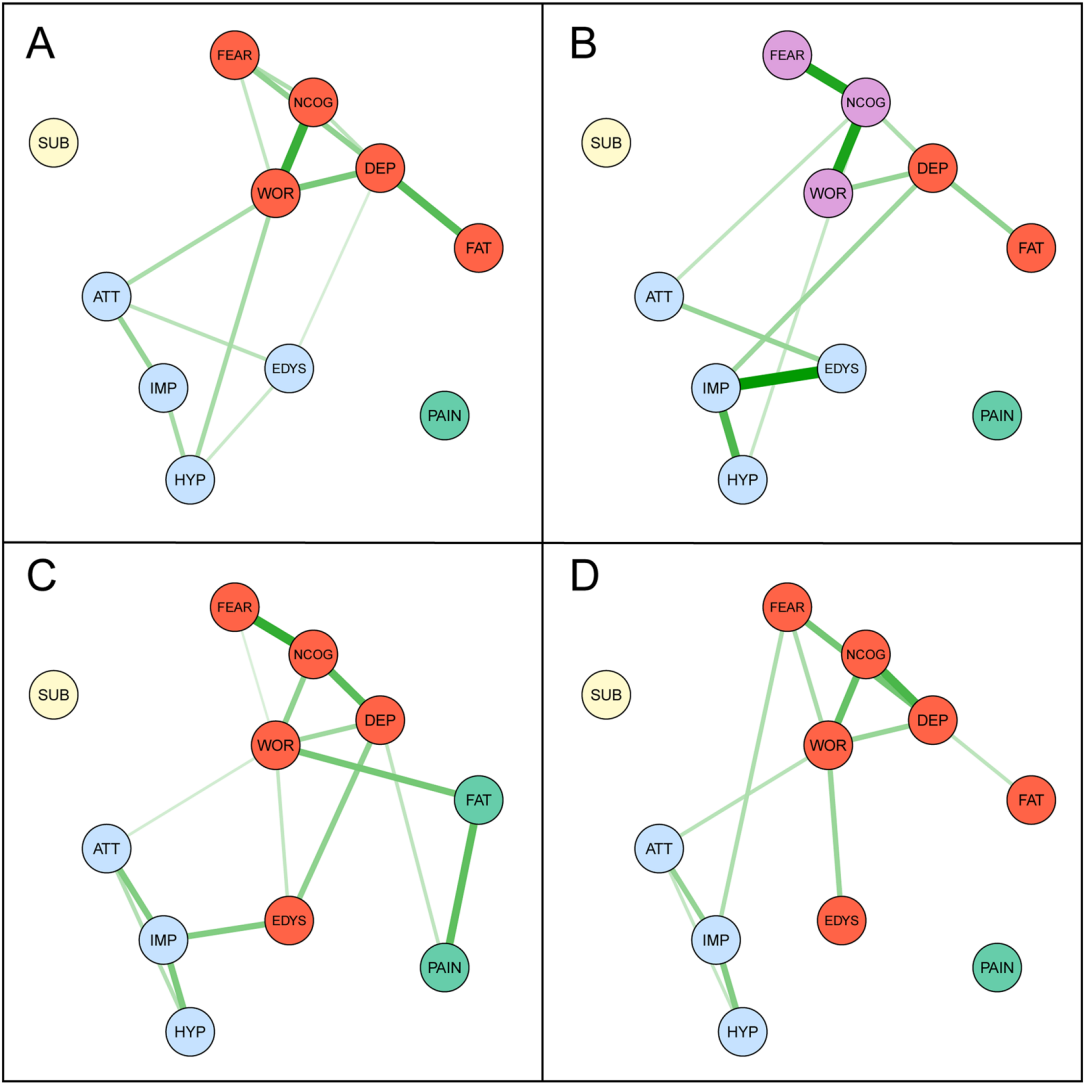
Network structure of symptom domains in four severity groups: (**A)** = CIDI subthreshold, (**B)** = CIDI lifetime only, (**C)** = CIDI 12mo-no-treatment, (**D)** = CIDI 12mo-yes-treatment. The network layout is kept constant (using average layout based on the four networks) to facilitate comparison. Symptom domains function as nodes; DEP indicates *Core depressive symptoms*; NCOG, *Negative cognitions about oneself*; FAT, *Fatigue*; FEAR, *Fearfulness*; PAIN, *Medically unexplained pain*; WOR, *Worrying*; IMP, *Impulsivity*; ATT, *Attention problems*; HYP, *Hyperactivity/restlessness*; EDYS, *Emotion dysregulation*; SUB, *Substance use*. Edges reflect the strength of pairwise symptom domain interrelations. To enable comparison of edge strength across networks, the strongest edge identified across all networks is set as maximum edge size. Thicker/darker edges imply stronger associations; green (solid) paths represent positive associations. Sub-communities identified by walktrap and edge-betweenness algorithms are indicated by different colors; domains with the same color belong to the same community.

We estimated a network for each severity group (see Fig. 1), in which symptom domains acted as nodes, and the pairwise partial associations between symptom domains as edges. To facilitate visual comparison, we plotted the networks as having an identical layout based on the average position of nodes across four networks. Thicker edges represent stronger associations. The overlapping 95% confidence intervals obtained with our stability analyses suggest caution in interpreting the relative strength of the interrelations (Supplementary Figs. S1–S4). The correlations of edge weights ranged from *r*_*s*_ = 0.33 for the association between networks of ‘CIDI Lifetime only’ and ‘12mo and treatment’, to *r*_*s*_ = 0.67 for the association between networks of ‘Subthreshold’ and ‘12mo and treatment’. All pairwise correlations are listed in Table 2. The Jaccard Index (see Supplementary Table S5) for each pairwise comparison of the unweighted networks showed the same pattern as the correlation coefficients.

**Table 2:** Spearman correlations of network edge weights across severity groups.

	G1	G2	G3
G2	0.49		
G3	0.47	0.39	
G4	0.67	0.33	0.53

Note: G1: CIDI subthreshold, G2: CIDI lifetime only, G3: CIDI 12mo-no-treatment, G4: CIDI 12mo-yes-treatment.

### Network comparison

#### Global strength

Pairwise comparisons of the groups using NCT (see Table 3) revealed that the four networks did not significantly differ in global strength: group 1 = 13.2, group 2 = 14.7, group 3 = 16.2 and group 4 = 12.5, respectively. The NCT for the unweighted networks, which can be found as Supplementary Table S6, showed the same results.

**Table 3:** Permutation results global strength Network Comparison Test.

	G1G2	G1G3	G1G4	G2G3	G2G4	G3G4
Global strength difference	1.51	2.98	0.76	1.46	2.26	373
P-value	0.76	0.55	0.85	0.77	062	0.41

Note: G1: CIDI subthreshold, G2: CIDI lifetime only, G3: CIDI 12mo-no-treatment, G4: CIDI 12mo-yes-treatment.

#### Community detection

In the networks of all four groups’ the walktrap and edge-betweenness community algorithms identified a community structure (both algorithms returned the same results). The estimated modularity indices for the final community solution in each group were: group 1 *Q* = 0.28, group 2 *Q* = 0.36, group 3 *Q* = 0.34, and group 4 *Q* = 0.29. As the values for *Q* are close together in all groups, with two slightly above the 0.30 cut-off and two groups slight below, we report the final community structure solution for all groups. Four communities were detected in each network, except for the network of group 2 (CIDI lifetime only) in which five communities were detected. Communities are highlighted in the networks in Fig. 1. Visual examination of the community structure revealed some notable similarities and differences between groups. Groups were highly similar in which symptom domains were part of the same community. In all groups, for instance, the nodes ‘attention’, ‘hyperactivity’ and ‘impulsivity’ were interrelated and formed a community, which we labeled ADHD-community. In all networks, interrelations were present between the domains ‘core depression’, ‘negative cognitions’ and ‘worrying’, and with exception of group 2, these domains belonged to the same community. Likewise, with the exception of group 2’s network, all networks featured an edge between the domains ‘attention problems’ and ‘worrying’ which bridged the ADHD- and depression/anxiety-community. In three of the four groups ‘core depression’ and ‘fatigue’ were part of the same community. The symptom domains ‘pain’ and ‘substance use’ were not interrelated to other symptom domains in all groups, except for group 3 in which ‘pain’ featured an edge with ‘fatigue’. The clearest difference between groups concerned the symptom domain ‘emotion dysregulation’. In the two groups with lowest illness severity, this domain belonged to the ADHD-community, while emotion dysregulation in the two highest illness severity groups was part of the depression/anxiety community. Community detection based on the unweighted networks returned a different solution for both algorithms (see Supplementary Figs. S5, S6). This did not lead to a different overall conclusion.

## Discussion

In this study, we compared the structure of symptom domain interrelations between four independent groups with increasing illness severity based on a diagnostic interview and referral status. With this approach, we gained insight whether interrelations between symptoms differ along the illness severity continuum. After controlling for symptom severity differences, we identified a coherent structure of symptom domain clustering in the four severity groups. Global network strength invariance and similarities in the number and type of communities detected in the four groups indicated that similarities in symptom domain clustering between de groups exceeded the differences. This is in contrast to frequent suggestions in the literature that psychopathology with increasing illness severity manifests as increasingly specific.

The present study evaluated the structure of symptom domain interrelations using several measures, i.e., global strength, community detection and edge weight correlations. Considering global strength, we observed no differences between the severity groups in how strong symptom domains overall were interrelated. While non-specificity (symptom domains equally likely to be interrelated) would have been reflected in low global strength, this was not the case. Modularity values in the four groups lay closely together, with lowest modularity values for the least and most severe illness group. Modularity is a continuous measure, and it is unlikely that values slightly above and below the cut-off score of 0.30 warrant a qualitatively different conclusion. Indeed, the community structures in each group were rather similar between groups: i.e., the four severity groups differed little in which symptom domains belonged to each community. In all four groups, we observed interrelations between symptom domains associated with attention deficit hyperactivity disorder (ADHD), and interrelations between symptom domains of depression and anxiety. The latter was also observed in adults from a clinical sample^33^, and in a general population sample in which these disorders were modeled at different ages during childhood and mid-adolescence^34^. At the most detailed structural level (similarity in edges weights across groups) we observed small to moderate similarity between severity groups. Hence, while groups differed in terms of which specific symptom pairs were interrelated, combined the interrelations between all symptoms-pairs did amount to a common community structure regardless of illness severity. These findings thus challenge the hypothesis that less severe or less frequent psychopathology presents as less specific than more severe psychopathology. Additionally, finding a consistent structure seems to be in line with findings from developmental psychopathology, which suggest that psychopathology already manifests in specific dimensions during preschool and that this structure remains strikingly consistent during the subsequent years^9,35^.

Several considerations need to be taken into account when interpreting our results. A first consideration pertains to our sample which may differ in composition compared to help-seeking populations with subthreshold symptoms that present at early intervention centers and which have informed clinical staging models and the specificity hypothesis^5,36,37^. In our large representative sample, individuals were not selected based on help-seeking behavior, and experienced a variety of psychiatric problems in both type and severity. In terms of subthreshold psychopathology, a large number of individuals in our sample met criteria for subthreshold ODD and CD. Individuals experiencing externalizing problems may be less likely to seek help than individuals with subthreshold depressive or anxious symptoms, as this difference in help-seeking behavior is observed for clinical disorders^38^. As such, help-seeking populations may result in biased presentation of the non-specificity of complaints in the subthreshold population.

A second important point is that clinical observations are difficult to make independent of severity. The increasing-specificity hypothesis may have face validity in clinical practice since distinct psychiatric problems for which help is sought may (seemingly) stand in the foreground. Moreover, apparent specificity is also facilitated by the hierarchical structure of DSM that aims to arrive at a primary diagnosis. It is important to repeat that in empirical research when comparing groups at different stages of psychopathology, it is necessary to eliminate the effect of symptom severity differences between the stages. This, because the mean symptom severity in a group influences the variance (particularly in the presence of floor effects)^39^, which influences the covariance, which is the unit of analysis in the network approach. We are the first do so efficiently, by establishing different threshold in each severity group to determine the presence of a symptom and subsequently kept the variance of domains equal across groups.

In addition to similarities, we observed some shifts in symptom-domain clustering between lower and higher levels of illness severity. Of those shifts, emotion dysregulation was most prominent, being interrelated with different symptom domains depending on whether individuals had less or more severe psychopathology. In the two lower severity groups, emotion dysregulation belonged to the ‘ADHD’ community (incl. attention problems, hyperactivity, and impulsivity) while in the higher severity groups it shared community membership with depressive and anxiety symptom domains. In the most severe group, interrelations with symptom domains of the ADHD community were completely absent. A logical explanation for this finding would be that the specific items within the emotion dysregulation domain that associate with the ADHD items differ from the emotion dysregulation items that cluster with depression and anxiety. Therefore, we post hoc investigated this possibility. To this end, we checked whether the endorsement on these separate items changed with increasing illness severity. This was not the case, indicating that there does not seem to be ADHD-specific or mood disorder-specific emotion dysregulation. We can also rule out that the community switch resulted from ADHD being relatively less severe in higher severity groups; there was no decline in the ADHD symptom domains. The switch of emotion dysregulation to the depression-anxiety community in the highest illness severity group could therefore indicate that emotion dysregulation as a symptom domain is mostly experienced as part of internalizing psychopathology when severity of psychopathology is high.

A major strength of the present study was our transdiagnostic approach; including individuals experiencing a wide range of psychiatric (sub)threshold disorders, and focusing on a variety of symptom domains not necessarily belonging to one disorder. Both are essential when addressing differences and similarities between different levels of illness severity. An additional strength of our study is that all individuals completed the same broad-psychopathology problem scale. This scale did not involve a skip structure, and therefore no conditional dependency between symptom domains (i.e., inflated correlations due to symptom domains only being assessed if core domain is endorsed), as in some other studies using networks^40^.

Using network methodology as a tool in the current study warrants that we address the recent debate concerning the replicability and robustness of networks^41–43^. An extensive discussion of the critique on networks is beyond the scope of this paper; hence, we limit ourselves to the issues relevant to this paper. Global network characteristics like those reported in our study (i.e., global strength and clustering) were found to be consistent across methods and samples^41^, indicating that these measures are robust. Likewise, a recent network investigation also observed a stable network disorder structure in a sample measured across three time points^44^. The edge-weight accuracy analyses in this study however revealed wide and overlapping confidence intervals surrounding edge weights. This is not unique to this network study but more frequently seen in psychopathology network analyses. This can be a real concern for the robustness of network findings as edge weights usually form the basis for subsequent structural analyses (e.g., global strength, community detection, and centrality indices^21^). We therefore repeated all our analyses using only the information whether edges were present or absent. The findings based on these unweighted networks were highly similar to the original findings and resulted in the same overall conclusion, indicating the robustness of our results.

Our results need to be considered in light of certain limitations. First, we used illness severity groups derived from cross-sectional data as a proxy for the illness severity continuum as referred to in the staging model. As such, the current findings do not inform on within person processes of increasing illness severity over time. This would require data with sufficient time points per person (>100), which is not yet available. Future studies with empirical data on individuals that move through different severity levels are necessary for the longitudinal clinical staging perspective. Note however that longitudinal investigations addressing the current research question may involve other limitations, such as whether a general clinical staging model holds for all psychiatric diagnoses or whether disorder specific within-person staging models need to be investigated. A second limitation is that although the CIDI assesses a wide spectrum of psychiatric diagnoses, it does not assess the diagnoses autism spectrum disorder (ASD) and psychosis. Individuals with these diagnoses may therefore have ended up in the wrong severity group. Third, groups were of different sizes. Although sample size influences network sparsity^20^, it is unclear to what extent this has affected our results (i.e., one of the smallest group had most estimated edges).

A more general limitation of network studies is that the network structure is dependent on the number and type of nodes (and thus the questionnaires measuring these) that are included in the network. Simultaneously, the number of nodes that can be included is restricted by the fact that parameters increase rapidly with every additional node, which reduces statistical power. By modeling symptom domains rather than single symptoms, we tried to optimize the balance between the number of nodes in the network and the range of constructs covered. Additionally, using broader constructs such as symptom domains has been recommended as one approach to improve measurement of symptoms in psychopathology networks^41^. Finally, it allowed making the prevalences of the domains comparable across groups. Practical reasons such as some problem domains being too rare (e.g., panic, suicidal ideation) or not adequately assessed within the ASR (e.g., positive psychotic symptoms, obsessive-compulsive symptoms, mania) meant that not all domains could function as nodes in the network. Although some domains were thus not included, the chosen domains and current transdiagnostic approach were highly comprehensive as compared to previous network analyses considering a narrow range of comorbid disorders^34^. Nonetheless, future studies need to corroborate our findings by including an even wider range of symptoms and disorders.

To conclude, the current study compared four frequently observed psychopathology severity groups in terms of the interrelations between symptom domains to investigate whether mild psychopathology manifests as less specific than more severe psychopathology. In four groups with increasing illness severity, we observed a rather consistent structure of symptom domain clustering which was invariant in global strength and in which we observed a distinct prototypical ADHD symptom domain cluster and a mood/anxiety domain cluster. Our findings suggest that subthreshold psychopathology does not manifest as less specific than more severe psychopathology.

## Supplementary information

Supplementary materials

## Data Availability

Data are from the TRacking Adolescents’ Individual Lives Survey (TRAILS). Readers may contact trails@umcg.nl to request the data. Information about the specific conditions under which the data are available can be found on www.trails.nl/en/hoofdmenu/data/ data use.
