# Supplementary material for: Specificity of psychopathology across levels of severity: a transdiagnostic network analysis
Source: Sci Rep. 2019 Dec 4;9:18298. doi: 10.1038/s41598-019-54801-y (PMC6892855; doi:10.1038/s41598-019-54801-y)
Supplement: Supplementary file 1 — Supplementary materials [file 41598_2019_54801_MOESM1_ESM.pdf]

SUPPLEMENTARY MATERIALS

Specificity of psychopathology across levels of severity: a transdiagnostic network analysis

Robin N. Groen, Marieke Wichers, Johanna T.W. Wigman, Catharina A. Hartman

Content:

1. Additional sample information pg. 2
2. Included CIDI diagnoses pg. 2
3. Network estimation pg. 2
4. Community algorithms pg. 3
5. Table S1 pg. 4
6. Table S2 pg. 8
7. Table S3 pg. 11
8. Table S4 pg. 12
9. Table S5 pg. 13
10. Table S6 pg. 14
11. Figure S1-S4 pg. 15
12. Figure S5-S6 pg. 19
13. References pg. 21

1. Additional sample information

TRAILS comprises a population-based cohort (TRAILS PC), which started in 2001, and a clinic-referred cohort (TRAILS CC) with the same instruments and timing of follow-up waves, starting in 2004. TRAILS participants were recruited from five municipalities in the North of the Netherlands, which included both urban and rural areas. The majority (86.5%) of the sample was of Dutch ethnicity. Prior to each assessment wave of TRAILS, informed consent was obtained from the adolescents (at the earlier waves also from the parents). An extensive description of the sampling procedures for TRAILS and TRAILS CC have been published elsewhere (Huisman et al., 2008; de Winter et al., 2005).

In the present study, data collected during the fourth assessment wave (T4) was used. For TRAILS PC T4 ran from October 2008 until September 2010, and 1881 adolescents (52 % female) participated (84% retention rate) with a mean age of 18.6 years (SD 0.6). For TRAILS-CC T4 ran from September 2012 until April 2014, and 422 adolescents (mean age 18.7±0.7 years, 34% females) of this cohort participated (78% retention rate). Data was pooled from both cohorts, to ensure the sample included a wide range of problem severity (i.e., both subclinical and clinical levels of psychopathology).

2. Included CIDI diagnoses

Included CIDI diagnoses were: mood disorders (major depressive disorder, dysthymic disorder, and bipolar disorder I and II), anxiety disorders (panic disorder, agoraphobia, social phobia, specific phobia, generalized anxiety disorder, separation anxiety disorder, and obsessive–compulsive disorder), behavioral disorders (attention-deficit/hyperactivity disorder, oppositional defiant disorder, and conduct disorder), substance use disorders (alcohol abuse/dependence, drug abuse/dependence) and pathological gambling.

3. Network estimation

Networks were estimated with the elasso procedure, which has been developed for the analysis of binary data and is based on the Ising model (van Borkulo et al., 2015). Associations between symptoms are identified through an iterative process of regularized logistic regression and model selection based on the Extended Bayesian Information Criterion (EBIC). Regularization occurs at two stages during elasso. A logistic regression is applied to each node (i.e., each node is regressed on all other nodes in the network) to investigate which nodes are part of the neighborhood (nodes that share an edge with that node) of that first node. Regularization during this stage occurs with *lasso* (least absolute shrinkage and selection operator); simply put, a penalization parameter ensures that spurious edges are set small to zero. By applying various values of the penalization parameter to the data the neighborhood selection is optimized. As this is done for the logistic regression of each node it results in multiple neighborhood selections. Next, an information criterion (EBIC in this case) is used to establish which set of neighborhoods constitutes the best fitting

model. During the model selection, a second regularization can be applied because a hyperparameter gamma (y) is incorporated in the EBIC. Larger values of gamma correspond to stronger penalization. If gamma is zero, no prior knowledge concerning the neighborhood set in the network is assumed, and no penalization is applied. We set gamma to zero. Following this procedure, we obtain the regression coefficients belonging to the best set of neighborhoods. As each node in the network is regressed on all other nodes, it can occur that an edge exist from *node a* to *node b* but not from *node b* to *node a*. The Ising model, however, only allows for undirected (bidirectional) nodes. We applied the so-called AND rule which holds that an edge between two nodes is only considered present if estimates for both edges (from *node a* to *b* and from *node b* to *a*) are nonzero. The weight of the bidirectional edge is the mean of both edges.

5. Community algorithms

The walktrap algorithm uses short random walks on a graph to detect communities (Pons & Latapy, 2005). It assumes that random walks within a graph should get “trapped" within the communities. The edge betweenness algorithm utilizes the betweenness centrality of edges, which reflects the number of times that an edge is part of the shortest paths between node pairs in a network (Girvan & Newman, 2002). High betweenness is often a sign that an edge is a bridge edge connecting two parts of the network. The algorithm removes edges sequentially based on the highest betweenness centrality to detect subnetworks (communities).

Table S1 – subthreshold disorders definitions

Disorder	DSM IV criteria	Definition used in paper	Definition used in other papers:
**General rules: criteria that concern functioning, or the disorder not being the result of another axis-1 disorder, or substance use are not considered for subthreshold status.**			
Obsessive Compulsive Disorder	A. Either obsessions or compulsions Obsessions as defined by (1), (2), (3), and (4):(1) recurrent and persistent thoughts, (2) the thoughts, impulses, or images are not simply excessive worries about real-life problems 3) the person attempts to ignore or suppress such thoughts, impulses, (4) the person recognizes that the obsessional thoughts, impulses, or images are a product of his or her own mind. Compulsions as defined by (1) and (2):(1) repetitive behaviors (2) the behaviors or mental acts are aimed at preventing or reducing distress or preventing some dreaded event or situation; B. At some point during the course of the disorder, the person has recognized that the obsessions or compulsions are excessive C. The obsessions or compulsions cause marked distress, are time consuming (take more than 1 hour a day), or significantly interfere with the person's normal routine, occupational (or academic) functioning D) not only restricted to other axis I disorder E) not due to substance use	- Criteria A + B<br>- Criteria A + C	De Bruijn et al., 2010 - subthreshold cases: subjects with obsessions and/or compulsions (the DSM-III-R A criterion) who did not meet the full criteria for OCD
Generalized Anxiety Disorder	A. Excessive anxiety and worry (apprehensive expectation), occurring more days than not for at least 6 months, about a number of events or activities (such as work or school performance).B. Person finds it difficult to control worry. C. The anxiety and worry are associated with three (or more) of the following six symptoms (with at least some symptoms present for more days than not for the past 6 months).1)restlessness2)fatigued3)concentrating difficulty4)irritability5)muscle tensions6)sleep disturbance D)worry not confined to other axis 1 disorder E)impaired functioning F)not due to substance	- Criteria: A (full: 6 months) + B + C (2 symptoms)<br>- Criteria: A (half: 3 months) + B + C (all symptoms)	Karsten et al., 2009 - subthreshold cases: excessive fear or worry about two or more life situations, difficult to control. At least 6 months with one to four physical of vegetative symptoms of anxiety.<br>Burstein et al., 2014 - subthreshold cases: relaxed duration of 3 months (all other criteria seem to apply).
Agoraphobia	A) anxiety about being in places or situations from which escape might be difficult (or embarrassing) or in which help may not be available in the event of having an unexpected or situationally predisposed Panic Attack or panic-like symptoms. Agoraphobic fears typically involve characteristic clusters of situations that include being outside the home alone; being in a crowd, or standing in a line; being on a bridge; and traveling in a bus, train, or automobile. B) The situations are avoided or else are endured with marked distress or with anxiety about having a Panic Attack or panic-like symptoms, or require the presence of a companion. C) The anxiety or phobic avoidance is not better accounted for by another mental disorder,	- Criteria: A (fear or anxiety in 1 place) + B	Heun et al. 2000 & Karsten et al., 2009 - subthreshold cases: Fear or anxiety of at least 2 places or situations from which it is difficult to leave (criteria A), however the individual endorses no avoidance behaviors of the feared situations (criteria B).

Social Anxiety Disorder	A. A marked and persistent fear of one or more social or performance situations in which the person is exposed to unfamiliar people or to possible scrutiny by others. B. Exposure to the feared social situation almost invariably provokes anxiety, which may take the form of a situationally bound or situationally predisposed Panic Attack. C. The person recognizes that the fear is excessive or unreasonable. Note: In children, this feature may be absent. D. The feared social or performance situations are avoided or else are endured with intense anxiety or distress. E) interference with normal routine F)duration at least 6 months G)not due to other disorder	*A + B + C<br>*A + C + D<br>*A + B + D	Karsten et al., 2009 - subthreshold cases: Persistent fear of situations in which the person is exposed to social interactions, for fear of acting in a humiliating or embarrassing way. Fear is recognized as excessive or unreasonable. Lack of avoidance.
Specific Phobia	A. Marked and persistent fear that is excessive or unreasonable, cued by the presence or anticipation of a specific object or situation (e.g., flying, heights, animals, receiving an injection, seeing blood).<br>B. Exposure to the phobic stimulus almost invariably provokes an immediate anxiety response, which may take the form of a situationally bound or situationally predisposed Panic Attack. C. The person recognizes that the fear is excessive or unreasonable. D. The phobic situation(s) is avoided or else is endured with intense anxiety or distress. E) interference with normal routine F)duration at least 6 months G)not due to other disorder	*A + B + C<br>*A + C + D<br>*A + B + D	Grenier et al. 2012 – subthreshold cases: subjects reporting at least one irrational fear not meeting the ‘‘excessiveness’’ and the ‘‘avoidance’’ criteria or not causing significant impairment in functioning
Panic Disorders	A. Both (A1) and (A2): (1) recurrent unexpected Panic Attacks, (2) at least one of the attacks has been followed by 1 month (or more) of one (or more) of the following: (a) persistent concern about having additional attacks(b) worry about the implications of the attack or its consequences (e.g., losing control, having a heart attack, "going crazy") (c) a significant change in behavior related to the attacks B) yes/no agoraphobia C) not due to substance, D) not due to other disorder	- Criterion A1<br>- Criterion A2	Roberts et al., 2015 : subthreshold cases: had a panic attack
ADHD	A. Either (1) or (2): (1) six (or more) of the following symptoms of inattention (2) six (or more) of the following symptoms of hyperactivity/impulsivity B. Some hyperactive-impulsive or inattentive symptoms that caused impairment were present before age 7 years C. Some impairment from the symptoms is present in two or more settings (e.g., at school [or work] and at home). D. There must be clear evidence of clinically significant impairment in social, academic, or occupational functioning. E. not because of other diagnosis.	- Criteria: A (3 inattention symptoms) + B (age of onset 12 years) + C<br>- Criteria: A (3 hyperactivity/impulsivity symptoms) + B (age of onset 12 years) + C	Roberts et al., 2015 : three inattention or three hyperactivity/impulsivity criteria;

Mania	A. A distinct period of abnormally and persistently elevated, expansive, or irritable mood, lasting at least 1 week (or any duration if hospitalization is necessary). B. During the period of mood disturbance, three (or more) of the following symptoms have persisted four if the mood is only irritable) and have been present to a significant degree: 1)inflated self-esteem 2)decreased sleep 3)more talkative 4)flight of ideas 5)distractibility 6)increase in goal directed behavior 7)excessive involvement in pleasurable activities that have a high potential for painful consequences C) no mixed episode D)severe impairment E)not due to substance	- Criteria: A (half – 4 days) + B (full – 4 symptoms)<br>- Criteria: A (full – 7 days) + B (half -2 symptoms)	Lewinsohn et al., 2000: subthreshold cases: distinct period of abnormally and persistently elevated, expansive, or irritable mood, in addition to having one or more manic or hypomanic symptoms
Major Depressive Disorder	A. Five (or more) of the following symptoms have been present during the same 2-week period and represent a change from previous functioning; at least one of the symptoms is either (1) depressed mood or (2) loss of interest or pleasure. 1) depressed mood 2) markedly diminished interest or pleasure 3) significant weight loss 4) insomnia or hypersomnia 5) psychomotor agitation or retardation 6) fatigue or loss of energy nearly 7) feelings of worthlessness or excessive or inappropriate guilt 8) diminished ability to think or concentrate 9) recurrent thoughts of death (n B. The symptoms do not meet criteria for a Mixed Episode. C. The symptoms cause clinically significant distress or impairment in social, occupational, or other important areas of functioning. D. The symptoms are not due to the direct physiological effects of a substance or a general medical condition. E. The symptoms are not better accounted for by Bereavement,	- Criteria A - 2 weeks + 3 symptoms (of which one is depressed mood/loss of pleasure)<br>- Criteria A – 1 week + 5 symptoms<br>- Minor depression (CIDI diagnosis)<br>- Recurrent brief depression (CIDI diagnosis)	Angst et al., 1997; Cuipers et al., 2004: Presence of less than five depressive symptoms for more than two weeks<br>Lewinsohn et al. 2004: three symptoms, minimum duration of 1 week.<br>Angst et al., 1997: 5 or more symptoms, less than two weeks
Oppositional Defiant Disorder	A. A pattern of angry/irritable mood, argumentative/defiant behavior, or vindictiveness lasting at least 6 months as evidenced by at least four symptoms from any of the following categories, and exhibited during interaction with at least one individual who is not a sibling. B. The disturbance in behavior is associated with distress in the individual or others in his or her immediate social context (e.g., family, peer group, work colleagues), or it impacts negatively on social, educational, occupational, or other important areas of functioning. C) not due to other disorder	- Criterion A (full)<br>- Criterion A (half – 2 symptoms) + B	Angold & Costello, 1996: subthreshold cases: children with two or more ODD symptoms involving some psychosocial impairment, such as interference with parental or peer relationships.<br>Roberts et al., 2015: subthreshold cases - meeting 2 ODD criteria
Conduct Disorder	A. A repetitive and persistent pattern of behavior in which the basic rights of others or major age-appropriate societal norms or rules are violated, as manifested by the presence of at least three of the following 15 criteria in the past 12 months from any of the categories below, with at least one criterion present in the past 6 months: B) The disturbance in behavior causes clinically significant impairment in social, academic, or occupational functioning C) individuals is age 18 years or older, criteria are not met for antisocial PD	- Criterion A (full)<br>- Criterion A (half – 2 symptoms) + B	Doyle et al., 2003 & Lewinsohn et al., 2004;Roberts et al. 2015: defined as two instead of three CD symptoms
Gambling Disorder	A. Persistent and recurrent maladaptive gambling behavior as indicated by five (or more) of the following	- Criterion A (half- 3 symptoms)	Cunningham-Williams et al. 2009 - subthreshold gambling are those who only meet 1 to 3 criteria.

Substance Dependence	A maladaptive pattern of substance use, leading to clinically significant impairment or distress, as manifested by three (or more) of the symptoms, occurring at any time in the same 12-month period.	- Criterion A (half - 2symptoms)	Pollock & Martin, 1999: Subthreshold substance use disorder was defined as one or two symptoms of any substance dependence (excluding alcohol and tobacco)
Alcohol Dependence	(A) A maladaptive pattern of drinking, leading to clinically significant impairment or distress, as manifested by three or more of the symptoms occurring at any time in the same 12-month period.	- Criterion A (half - 2 symptoms)	Rohde, Lewinsohn & Seeley, 1996: one or more symptoms of alcohol dependence and not had abuse

Table S2: Symptom domains based on multiple ASR items with varying thresholds for the diagnostic groups

Construct	Items	Group	Rule
**1. Core depression symptoms**	c4ar51= There is very little that I enjoy.	4	1 item >2 and 1 item >1
	c4ar85= I am unhappy, sad, or depressed	3	1 item >2
	c4ar12= I cry a lot.	2	1 item above >2 or 3 items >1
		1	1 item above >2 or 2 items each >1
**2. Negative cognitions about self**	c4ar44 = I feel too guilty	4	2 items >2 or 1 item>2 and 3 items >1
	c4ar88= I feel that I can't succeed	3	1 item >2 and 1 item >1
	c4ar30= I feel worthless or inferior	2	1 item >2 or 4 items >1
	c4ar61=I am self-conscious or easily embarrassed	1	1 item >2 or 3 items >1
**3.Fatigue**	c4ar84= I don't have much energy	4	2 items >2
	c4ar46= I feel tired without good reason	3	2 items >2
		2	1 item >2 and 1 item >1
		1	1 item >2
**4.Worry**	c4ar19=I often worry about my worries[1]	4	3 items >2 and 1 item >1
	c4ar92= I worry a lot	3	3 items>2 or 2 items > 2 and 2 items >1
	c4ar62=I worry about my family	2	3 items>2 or 2 items > 2 and 2 items >1
	c4ar7=I can't get my mind of certain thoughts	1	2 items > 2 and 1 item >1 or 1 item >2 and 3 items >1
**5. Fearfulness**	c4ar38= I am nervous or tense	4	1 item>2 and 1 item >1

[1] This is an English translation of ASR item 22 as it is listed in the Dutch version of ASR: "Ik maak mij vaak zorgen om mijn zorgen", and therefore slightly deviates from the original item in English: "I worry about my future".

	c4ar42=I am too fearful or anxious	3	1 item >2
		2	1 item >2
		1	1 item >2 or both items >1
**6.Unexplained Pain symptoms**	c4ar48a = Aches or pains (**not** stomach or headaches)	4	1 item >2 and 1 item >1
	c4ar48b = Headaches	3	1 item >2
	c4ar48f = Stomachaches	2	1 item >2 or 2 items >1
		1	1 item>1
**7.Impulsivity**	c4ar35= I am impulsive or act without thinking	4	2 item >2 or 1 item>2 and 2 items >1
	c4ar74= I rush into things without considering the risks	3	2 item >2 or 1 item>2 and 2 items >1
	c4ar98 = I am too impatient	2	1 item >2
		1	1 item >2 or 3 items >1
**8.Attention problems**	c4ar6= I have trouble concentrating or paying attention for long	4	4 items > 2 and 1 item >1 or 3 items > 2 and 3 items > 1
	c4ar14= I daydream a lot.	3	4 items > 2 or 3 items>2 and 2 items>1 or 2 items > 2 and 4 items >1
	c4ar89: I tend to lose things	2	3 items >2 and 1 item >1 or 2 items >2 and 3 items >1 or 1 item>2 and 5 items >1
	c4ar99= I am not good at details	1	3 items >2 or 2 items >2 and 2 items>1 or 1 item > 2 and 4 items > 1
	c4ar1= I am too forgetful	4	
	c4ar45 = I have trouble planning for the future		
**9. Hyperactivity**	c4ar8= I have trouble sitting still	4	2 items >2
	c4ar95= I feel restless or fidgety	3	1 item > 2 and 2 items >1 or 2 items >2
	c4ar78= I talk too much	2	2 items > 2 or 1 item >2 and 2 items > 1 or all 3 items >1
		1	1 item >2 or 3 items >1

**10.Emotion dysregulation**	c4ar72= I am stubborn, sullen, or irritable	4	1 item >2 and another >1
	c4ar80= I have a hot temper.	3	1 item >2
	c4ar2= I argue a lot	2	1 item >2
		1	1 item >2 or 3 items >1
**11. Substance use**	c4ar75= I drink too much alcohol or get drunk	4	1 item >2
	c4ar4= I use drugs (other than alcohol and nicotine) for nonmedical purposes (describe	3	1 item >2
		2	1 item >2 or 2 items >1
		1	1 item >2 or 2 items >1

Table S3 - Symptom domain prevalence within each outcome group after applying group specific thresholds for each symptom domain

Symptom domains prevalences *N* (%)	CIDI subthreshold (n= 494)	CIDI lifetime only (n= 204)	CIDI 12mo-no-treatment (n= 288)	CIDI 12mo-yes-treatment (n= 303)
Core depression	75 (15.2%)	24 (11.7%)	43 (14.8%)	41 (13.5%)
Negative cognitions about oneself	56 (11.4%)	34 (16.6%)	47 (16.2%)	45 (14.9 %)
Fatigue	45 (9.1%)	18 (8.8%)	22 (7.6%)	36 (11.9%)
Fearfulness	48 (9.8%)	20 (9.8%)	26 (8.9%)	38 (12.5%)
Medically unexplained pain	66 (13.4%)	20 (9.8%)	40 (13.7%)	42 (13.9%)
Worrying	47 (9.6%)	22 (10.7%)	30 (10.3%)	29 (9.6%)
Impulsivity	74 (15.0%)	26 (12.7%)	39 (13.4%)	50 (16.5%)
Attention problems	68 (13.8%)	27 (13.2%)	45 (15.5%)	39 (12.9%)
Hyperactivity/restlessness	74 (15.0%)	33 (16.1%)	35 (12.0%)	33 (10.9%)
Emotion dysregulation	42 (8.5%)	26 (12.7%)	34 (11.7%)	42 (13.5%)
Substance use	68 (13.8%)	29 (14.1%)	37 (12.7%)	45 (14.9%)

Table S4 - Levene's test for homogeneity of variance symptom domains after applying group specific thresholds for each symptom domain

Symptom domains	Variance G1	Variance G2	Variance G3	Variance G4	F	Df	P
Core depression	0.13	0.10	0.13	0.12	0.55	3	0.65
Negative cognitions about oneself	0.10	0.14	0.14	0.13	1.57	3	0.16
Fatigue	0.08	0.08	0.07	0.10	1.18	3	0.32
Fearfulness	0.09	0.09	0.08	0.11	0.84	3	0.47
Medically unexplained pain	0.12	0.09	0.12	0.12	0.78	3	0.51
Worrying	0.09	0.10	0.09	0.09	0.10	3	0.96
Impulsivity	0.13	0.11	0.12	0.14	0.64	3	0.59
Hyperactivity/restlessness	0.12	0.11	0.13	0.11	1.64	3	0.18
Attention problems	0.13	0.14	0.11	0.10	0.31	3	0.82
Emotion dysregulation	0.08	0.11	0.10	0.12	1.78	3	0.15
Substance use	0.12	0.12	0.11	0.13	0.20	3	0.90

Note: G1: CIDI subthreshold, G2: CIDI lifetime only, G3: CIDI 12mo-no-treatment, G4: CIDI 12mo-yes-treatment.

Table S5. Jaccard index of network edges across severity groups

	G1	G2	G3
G2	0.39		
G3	0.45	0.30	
G4	0.53	0.28	0.50

Note: G1: CIDI subthreshold, G2: CIDI lifetime only, G3: CIDI 12mo-no-treatment, G4: CIDI 12mo-yes-treatment.

Table S6. Permutation results global strength Network Comparison Test for unweighted networks

	G1G2	G1G3	G1G4	G2G3	G2G4	G3G4
Global strength difference	3	1	2	4	1	3
P-value	0.80	0.96	0.85	0.51	0.92	0.65

Note: G1: CIDI subthreshold, G2: CIDI lifetime only, G3: CIDI 12mo-no-treatment, G4: CIDI 12mo-yes-treatment.

Fig S1. Bootstrap edge accuracy plot – group 1: CIDI subthreshold

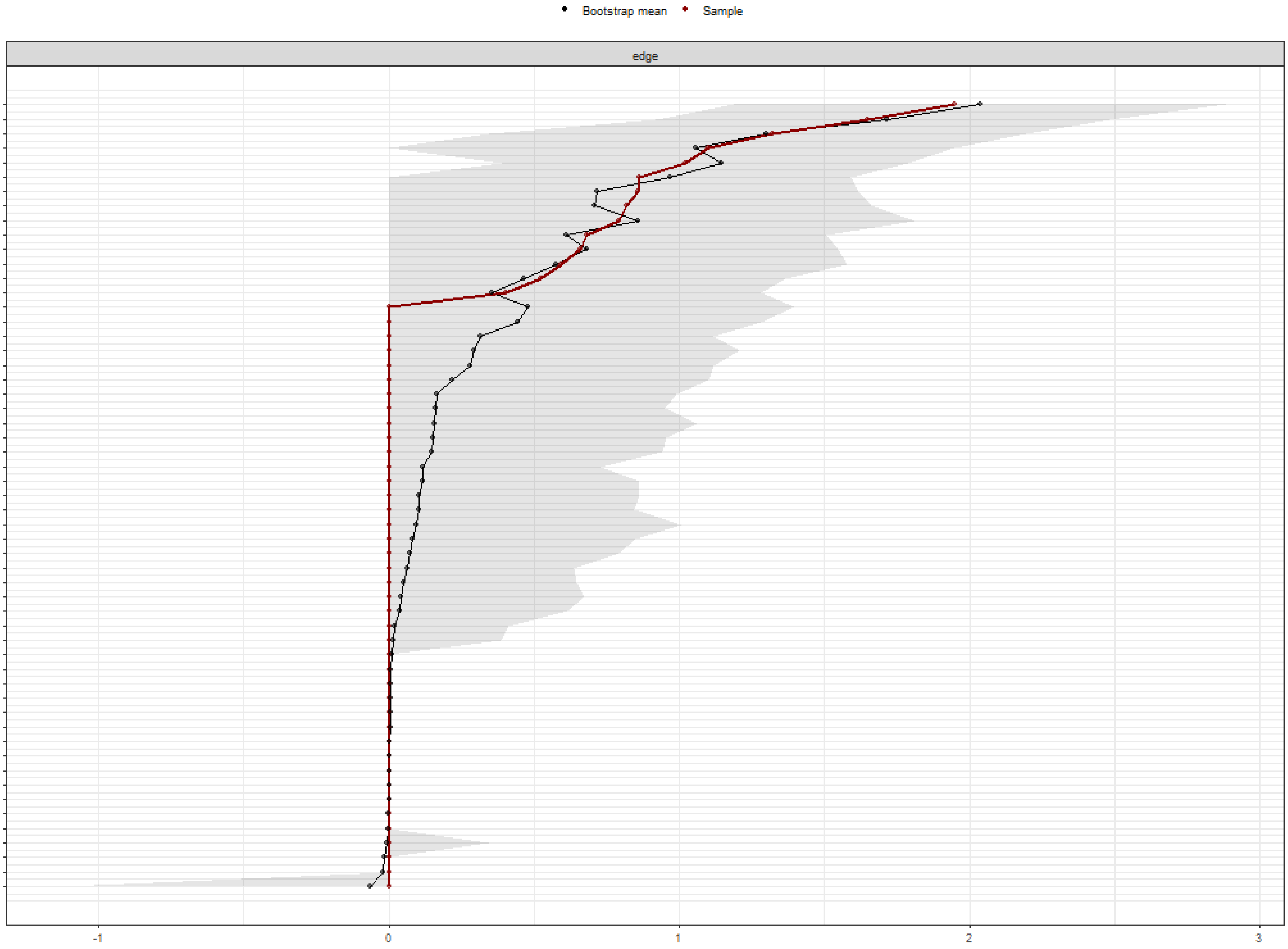

Fig S2. Bootstrap edge accuracy plot – group 2: CIDI lifetime only

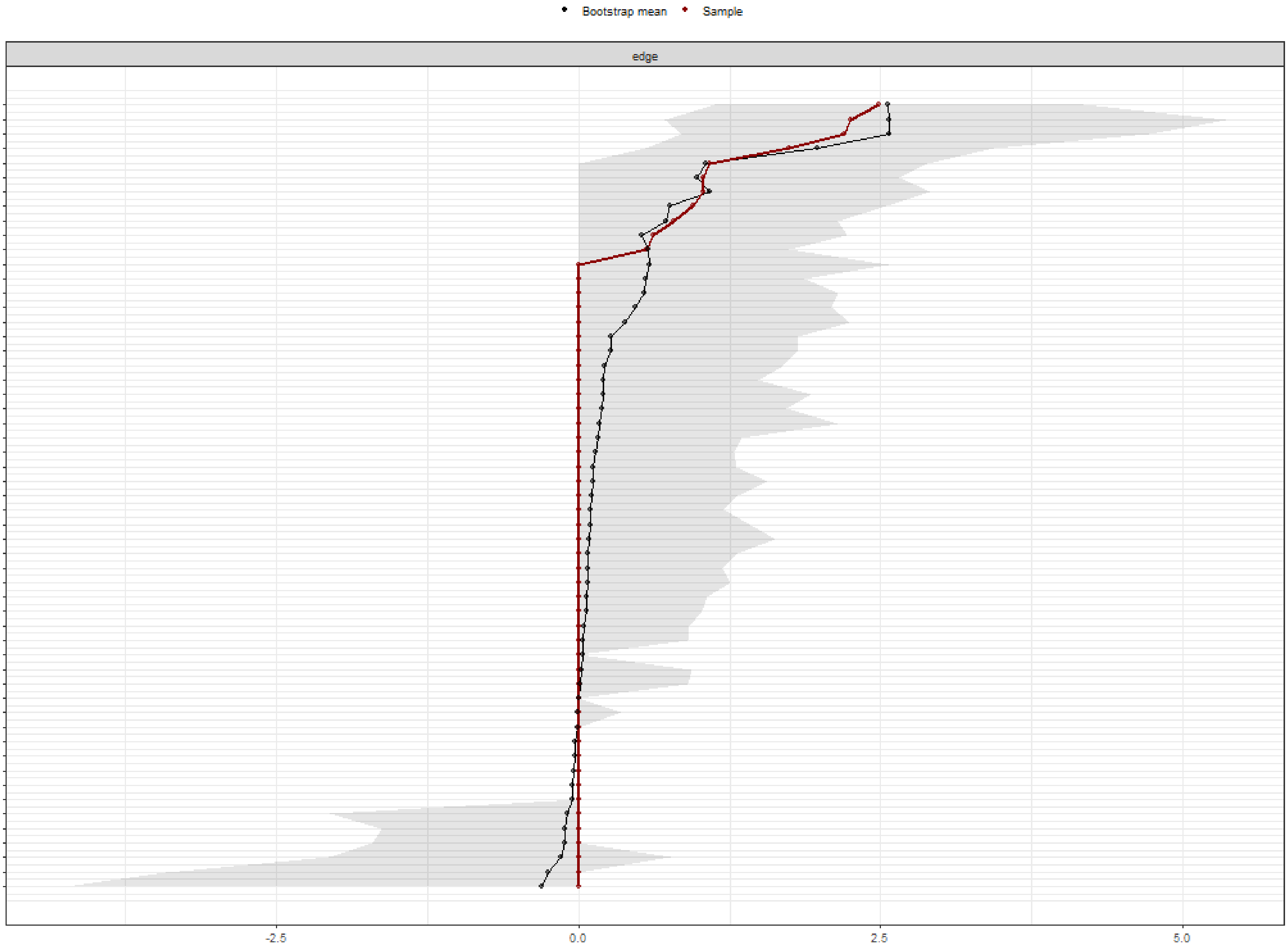

Fig S3. Bootstrap edge accuracy plot – group 3: CIDI 12mo-no- treatment

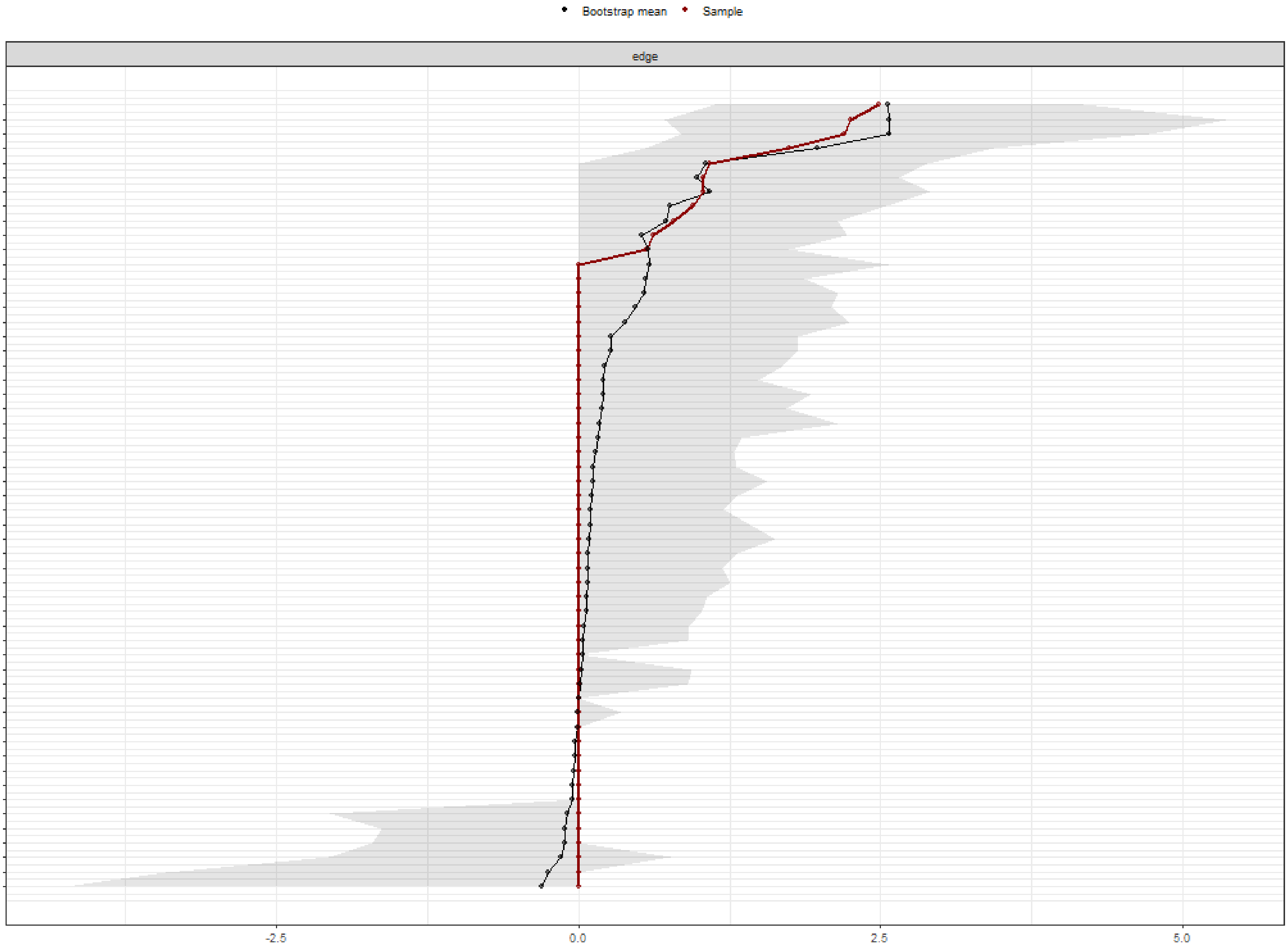

Fig S4. Bootstrap edge accuracy plot – group 4: CIDI 12mo-yes- treatment

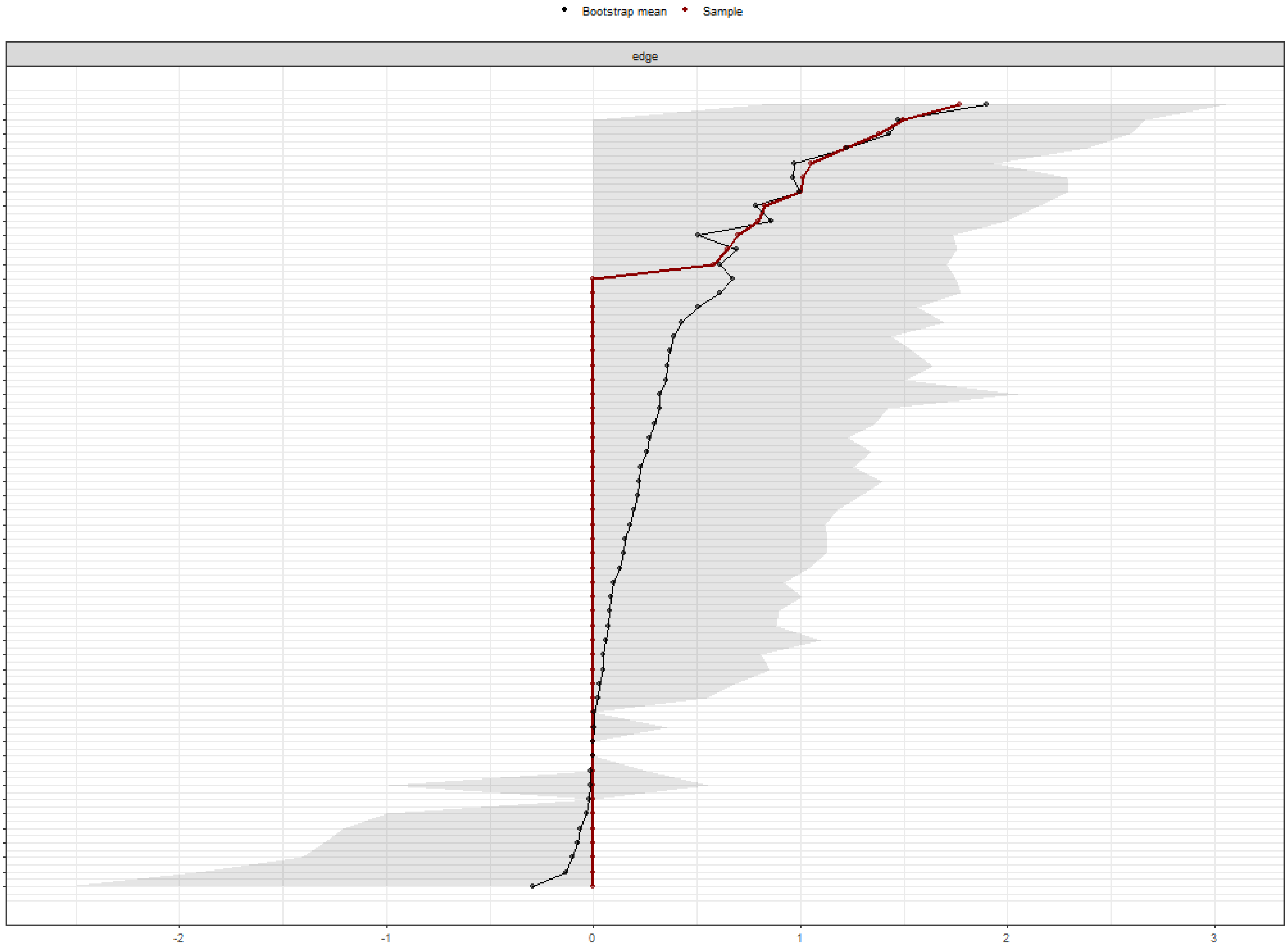

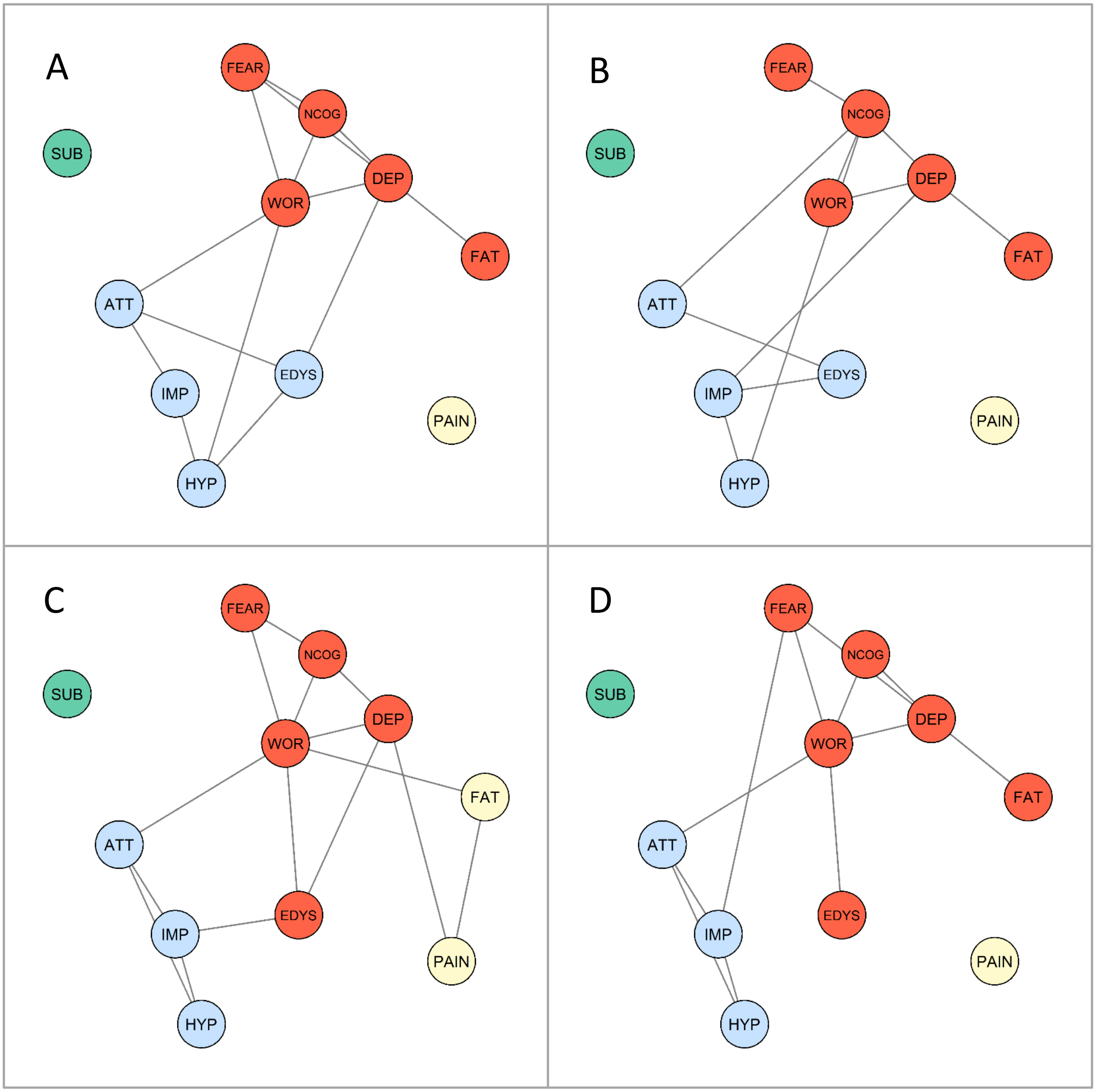

Figure S5. Community structure returned by the Walktrap algorithm based on the unweighted networks of the four severity groups: A = CIDI subthreshold, B= CIDI lifetime only, C= CIDI 12mo-no-treatment, D = CIDI 12mo-yes-treatment. Symptom domains function as nodes; DEP indicates *Core depressive symptoms*; NCOG, *Negative cognitions about oneself* ; FAT, *Fatigue*; FEAR, *Fearfulness*; PAIN, *Medically unexplained pain*; WOR, *Worrying*; IMP, *Impulsivity*; ATT, *Attention problems*; HYP, *Hyperactivity/restlessness*; EDYS, *Emotion dysregulation*; SUB, *Substance use.* Sub-communities are indicated by different colors; domains with the same color belong to the same community.

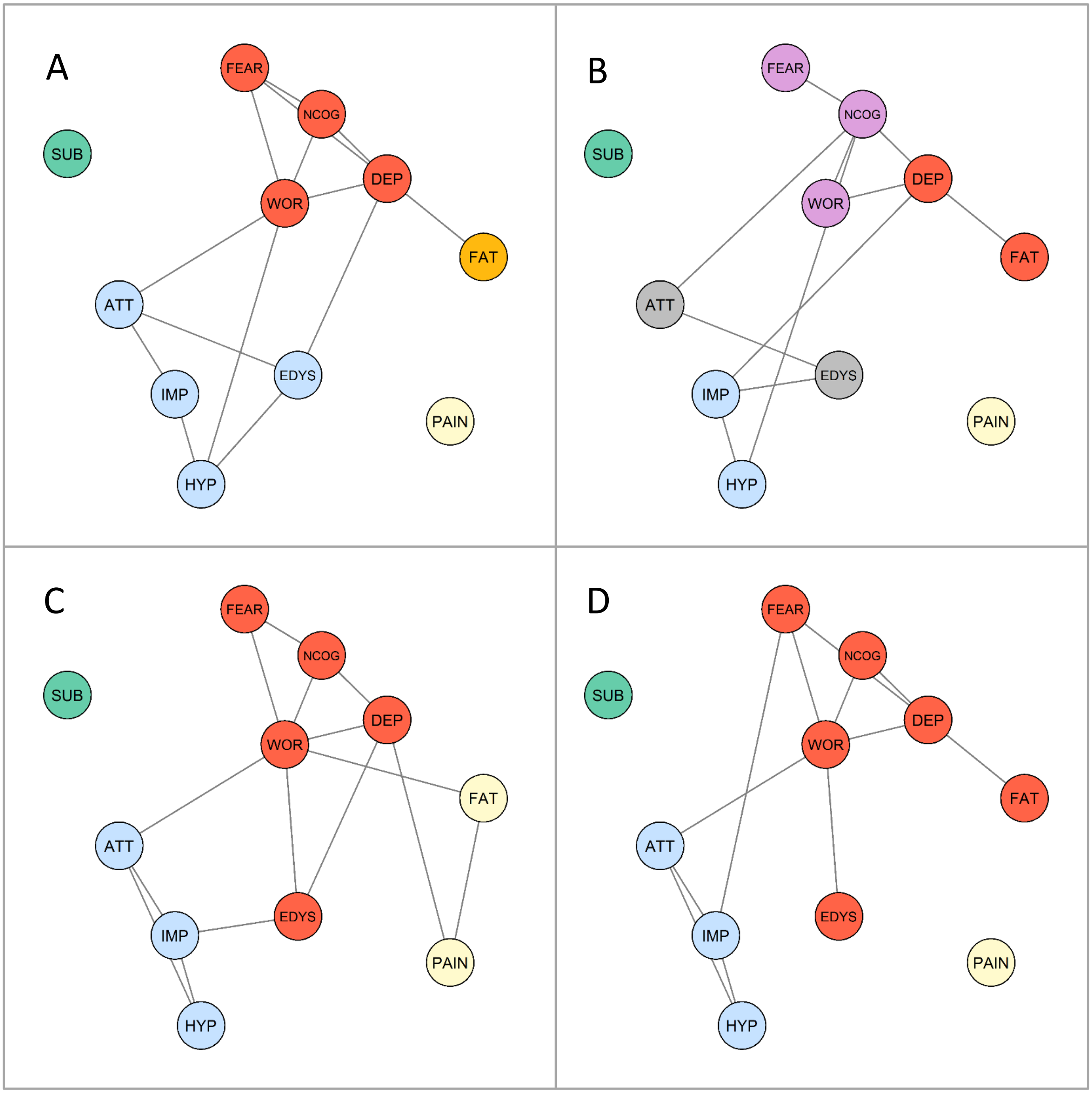

Figure S6. Community structure returned by the Edge-Betweenness algorithm based on the unweighted networks of the four severity groups: A = CIDI subthreshold, B= CIDI lifetime only, C= CIDI 12mo-no-treatment, D = CIDI 12mo-yes-treatment. Symptom domains function as nodes; DEP indicates *Core depressive symptoms*; NCOG, *Negative cognitions about oneself* ; FAT, *Fatigue*; FEAR, *Fearfulness*; PAIN, *Medically unexplained pain*; WOR, *Worrying*; IMP, *Impulsivity*; ATT, *Attention problems*; HYP, *Hyperactivity/restlessness*; EDYS, *Emotion dysregulation*; SUB, *Substance use.* Sub-communities are indicated by different colors; domains with the same color belong to the same community

References

Angold, A., & Costello, E. J. (1996). Toward establishing an empirical basis for the diagnosis of oppositional defiant disorder. *Journal of the American Academy of Child & Adolescent Psychiatry*, 35(9), 1205-1212.

Angst, J., & Merikangas, K. (1997). The depressive spectrum: diagnostic classification and course. Journal of affective disorders, 45(1-2), 31-40.

Burstein, M., Beesdo-Baum, K., He, J. P., & Merikangas, K. R. (2014). Threshold and subthreshold generalized anxiety disorder among US adolescents: prevalence, sociodemographic, and clinical characteristics. *Psychological Medicine*, *44*(11), 2351-2362.

De Bruijn, C., Beun, S., De Graaf, R., Ten Have, M., & Denys, D. (2010). Subthreshold symptoms and obsessive–compulsive disorder: evaluating the diagnostic threshold. *Psychological Medicine*, *40*(6), 989-997.

Cunningham-Williams, R. M., Gattis, M. N., Dore, P. M., Shi, P., & Spitznagel Jr, E. L. (2009). Towards DSM-V: considering other withdrawal-like symptoms of pathological gambling disorder. *International Journal of Methods in Psychiatric Research*, 18(1), 13-22.

Doyle, A. E., Biederman, J., Monuteaux, M., Cohan, S. L., Schofield, H. L. T., & Faraone, S. V. (2003). Diagnostic threshold for conduct disorder in girls and boys. *The Journal of Nervous and Mental Disease,* 191(6), 379-386.

Cuijpers, P., de Graaf, R., & van Dorsselaer, S. (2004). Minor depression: risk profiles, functional disability, health care use and risk of developing major depression. *Journal of Affective Disorders*, 79(1-3), 71-79.

Grenier, S., Potvin, O., Hudon, C., Boyer, R., Préville, M., Desjardins, L., & Bherer, L. (2012). Twelve-month prevalence and correlates of subthreshold and threshold anxiety in community-dwelling older adults with cardiovascular diseases. *Journal of Affective Disorders*, *136*(3), 724-732.

Heun, R., Papassotiropoulos, A., & Ptok, U. (2000). Subthreshold depressive and anxiety disorders in the elderly. *European Psychiatry*, *15*(3), 173-182.

Huisman, M., Oldehinkel, A. J., de Winter, A., Minderaa, R. B., de Bildt, A., Huizink, A. C., ... & Ormel, J. (2008). Cohort profile: The dutch 'TRacking adolescents' individual lives' survey'; TRAILS. *International Journal of Epidemiology,* 37(6), 1227-1235.

Karsten, J., Hartman, C. A., Smit, J. H., Zitman, F. G., Beekman, A. T., Cuijpers, P., ... & Penninx, B. W. (2011). Psychiatric history and subthreshold symptoms as predictors of the occurrence of depressive or anxiety disorder within 2 years. *The British Journal of Psychiatry*, *198*(3), 206-212.

Lewinsohn, P. M., Klein, D. N., & Seeley, J. R. (2000). Bipolar disorder during adolescence and young adulthood in a community sample. *Bipolar Disorders*, 2(3p2), 281-293.

Lewinsohn, P. M., Shankman, S. A., Gau, J. M., & Klein, D. N. (2004). The prevalence and co-morbidity of subthreshold psychiatric conditions. *Psychological Medicine*, 34(4), 613-622.

Pollock, N. K. & Martin, C. S. (1999). Diagnostic orphans: adolescents with alcohol symptomatology who do not qualify for DSM-IV abuse or dependence diagnoses. *American Journal of Psychiatry* 156, 897–901.

Roberts, R. E., Fisher, P. W., Turner, J. B., & Tang, M. (2015). Estimating the burden of psychiatric disorders in adolescence: The impact of subthreshold disorders. *Social Psychiatry and Psychiatric Epidemiology*, *50*(3), 397-406.

Rohde, P., Lewinsohn, P. M. & Seeley, J. R. (1996). Psychiatric comorbidity with problematic alcohol use in high school adolescents. *Journal of the American Academy of Child and Adolescent Psychiatry,* 35, 101–109.

de Winter, A. F., Oldehinkel, A. J., Veenstra, R., Brunnekreef, J. A., Verhulst, F. C., & Ormel, J. (2005). Evaluation of non-response bias in mental health determinants and outcomes in a large sample of pre-adolescents. *European Journal of Epidemiology*, *20*(2), 173-181.
